# Indeno[1,2-*b*]thiophene End-capped Perylene Diimide: Should the 1,6-Regioisomers be systematically considered as a byproduct?

**DOI:** 10.1038/s41598-020-60012-7

**Published:** 2020-02-24

**Authors:** Pablo Simón Marqués, Francesco Tintori, José María Andrés Castán, Pierre Josse, Clément Dalinot, Magali Allain, Gregory Welch, Philippe Blanchard, Clément Cabanetos

**Affiliations:** 1 CNRS UMR 6200, MOLTECH-Anjou, University of Angers, 2 Bd Lavoisier, 49045 Angers, France; 2 Department of Chemistry, University of Calgary, 2500 University Drive N.W., Calgary, Alberta T2N 1N4 Canada

**Keywords:** Energy, Materials chemistry, Organic chemistry

## Abstract

Usually considered as a byproduct, the *1,6-*dibrominated PDI has rarely been functionalized for the preparation of electro-active conjugated molecules, particularly in the field of organic photovoltaics. In light of the literature, one can ask oneself: Does a *1,7*-isomer based functional molecule systematically perform better than its *1,6*-analogue? To answer this question, we report herein the synthesis and direct comparison of two indeno[1,2-*b*]thiophene (IDT) end-capped perylene diimide regioisomers (PDI) (*1,6* and *1,7*) used as non-fullerene acceptors in organic solar cells. It turned out that in our case, *ie*, when blended with the well-known PTB7-Th donor polymer, higher performance was reached for devices made with the *1,6*-analogue.

## Introduction

**Figure 1:**
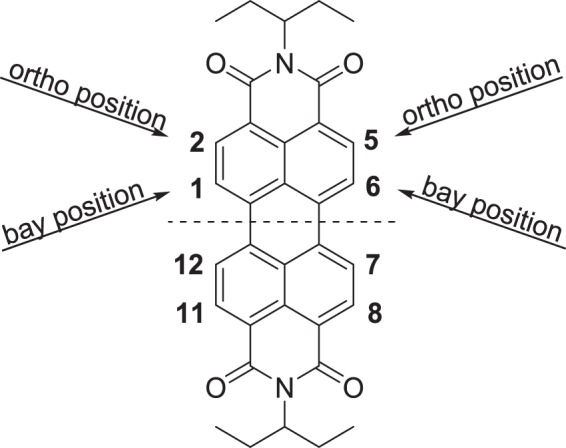
Molecular structure of the perylene diimide chromophore.

Major contributors to the recent resurgence of organic photovoltaics (OPVs)^1–3^, molecular non-fullerene acceptors (NFAs) have driven up power conversion efficiencies (PCEs), in a short amount of time, from “what a fullerene derivative does best”^4,5^ to more than 15%^6–8^. Although these records were reached with indacenodithiophene derivatives, perylene diimides (PDIs) have always held an important place among the electron transporting materials due to their excellent thermal, chemical and photochemical stabilities combined with their singular optical, redox and charge transport properties^9,10^. Nonetheless, the PDI extended π-conjugated structure typically suffers from an excessive π–π stacking tendency, forming micrometer-sized crystallites that are detrimental for an efficient charge separation in bulk heterojunction (BHJ) solar cells^11–14^. To tackle this problem, important efforts have been devoted to functionalize the peripheral positions, namely the bay (1,6,7,12) and *ortho* positions (2,5,8,11)^3,9,15–18^, leading to profuse design principles and above all, to a better understanding of the structure-property relationships (Fig. 1)^19–28^.

The key reaction, the dibromination of the bay positions, holds a special place by providing a precursor to many published molecular and macromolecular π-functional PDI based materials, namely the *1,7*-isomer^29^. Also generated during the reaction, but in smaller ratio, the *1,6*-isomer has not triggered such interest. Conversely, the latter has even motivated the development of purification procedures and methods since usually considered as an impurity^30^.

As a result, only limited studies aiming at comparing the properties of molecular architectures based on both regioisomers have been reported so far^31–33^, particularly in the field of OPVs. In fact, and to the best of our knowledge, only one paper can be found where the two bromo isomers (*1,6* and *1,7*) were functionalized either by triphenylamine (TPA) or benzodithiophene (BDT) moieties for the preparation of NFAs^34^. In all cases, best PCEs (*ca* 0.67%) were reached with the *1,7*-isomers once blended with different donor polymers and attributed to a better phase separation. Considering the scarcity of reported examples, it would be unthinkable to draw meaningful conclusions on the systematic use of one isomer over the other.

Consequently, to gain further insight into these differences, particularly from a charge transport point of view, the synthesis and direct comparison of two new indeno[1,2-*b*]thiophene (IDT) end-capped PDI regioisomers (*1,6* and *1,7*) is reported herein.

## Results and Discussion

**Scheme 1:**
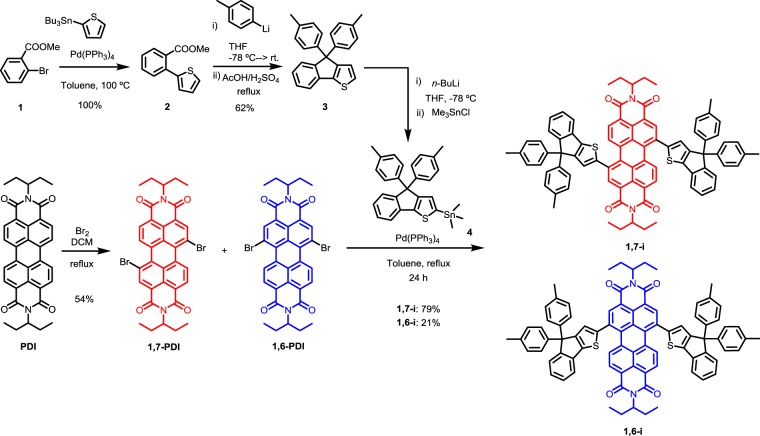
Synthetic route to **1,6-i** and **1,7-i**.

The synthetic route to both target regioisomers **1,7-i** and **1,6-i** is depicted in Scheme 1.

Stemming from the current high efficient NFAs^3,35,36^, the bulky IDT derivative (**4**), used herein to restrain the PDI aggregation in **1,7-i** and **1,6-i**, was first prepared in three steps starting with a Stille pallado-catalysed reaction between the methyl 2-bromobenzoate **1** and the 2-tributylstannylthiophene to afford compound **2**. The latter was then treated with *p*-tolyllithium to generate a benzyl alcohol intermediate that was subsequently cyclized under acidic conditions. The resulting indeno[1,2-*b*]thiophene **3** was finally stannylated in presence of *n*-BuLi and trimethyltin chloride. In parallel, a 3:1 ratio mixture of *1,7* and *1,6* dibrominated PDIs was efficiently prepared following a method reported by Rybtchinski & *al*.^37^ before being directly engaged in a final Stille cross-coupling reaction with the stannyl derivative **4** to afford the mixture of target isomers that were easily separated by column chromatography.

**Figure 2:**
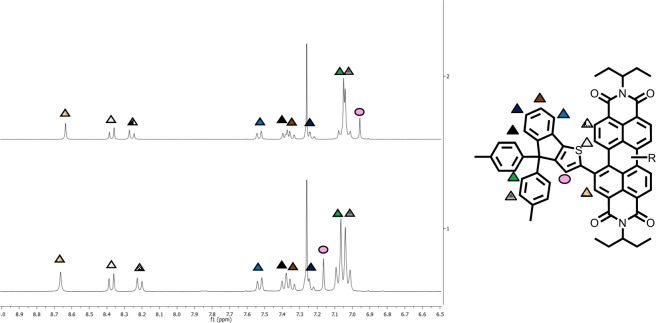
^1^H NMR (300 MHz) spectra of **1,6-i** (top) and **1,7-i** (bottom) in CDCl_3_ at 25 °C.

Analyzed by ^1^H NMR spectroscopy, the latter exhibit similar sets of signals with only subtle changes, as depicted in Fig. 2. In fact, only the singlet signal associated to the proton born by the thiophene ring showed a significantly different chemical shift at 6.96 ppm and 7.17 pm for **1,6-i** and **1,7-i** respectively (pink circle, Fig. 2).

**Figure 3:**
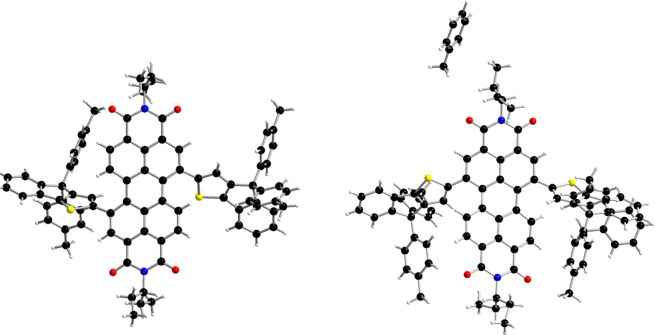
Molecular structures of **1,7-i** (left) and **1,6-i** (right) obtained by X-ray diffraction.

Although structural attribution can be deduced from the ^13^C NMR spectra, and more precisely from the number of aliphatic carbon that are symmetry dependent (Fig. S11), single crystals of both isomers were grown by the solvent evaporation method. While slow evaporation from dichloromethane was used for **1,7-i**, it is noteworthy that stable and suitable single crystals of its *1,6* couterpart were only obtained via slow evaporation from toluene. Beyond confirming the structure of each isomer (Fig. 3), X-ray diffraction analyses also revealed, in both cases, regardless of the grafting position, (*i)* relatively twisted PDI backbones with similar dihedral angles (of *ca* 19°) between the two subplanes and (*ii)* high torsion angles (>44°) of the PDI cores with the bulky IDT arms, hindering their aggregation and resulting in a good solubility in common organic solvents (Figs. S20–S24).

**Figure 4:**
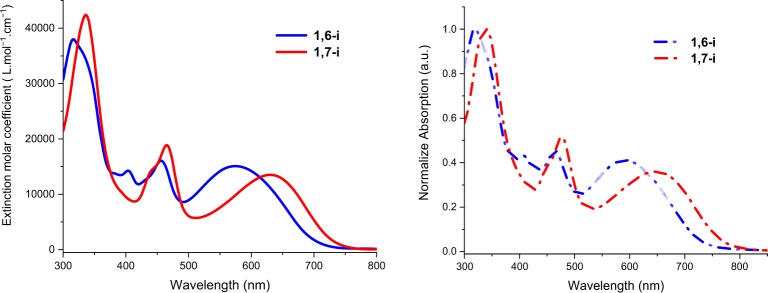
UV-Vis absorption spectra of **1,6-i** (blue line) and **1,7-i** (red line) in CH_2_Cl_2_ (left) and as thin film on glass (right).

The two isomers were analyzed by UV-visible spectroscopy revealing a strong absorption in the visible/near-infrared region in both solution and as thin films (Fig. 4). Optical data are gathered in Table 1.

**Table 1:** Optical data of **1,6-i** and **1,7-i** recorded in dichloromethane solutions (10^−6^ M) and on glass sheets.

Compound	λ_max_^ABS^ (nm) in DCM	ε (M^−1^ cm^−1^)	λ_max_^ABS^ (nm) in film	E_g_^opt^ (eV)
**1,6-i**	573	16000	591	1.66
	456	17000	465	
	316	41000	318	
**1,7-i**	630	14000	650	1.56
	465	17000	478	
	336	41000	338	

As expected, similar patterns were recorded showing three maxima at *ca* 320 nm, 460 nm and in the 550–650 nm region assigned to π-π* transitions localized on each building blocks, *ie*, on the IDT and on the PDI units, and to a charge transfer (CT) transition from the electron rich substituents (IDT) to the electro deficient central core (PDI) respectively. Nonetheless, comparison of the spectra clearly highlights the impact of the grafting positions on the electronic properties since all absorption bands of **1,7-i** are significantly red shifted with respect to those of **1,6-i**, particularly in the low energy region (CT band) resulting in a lower optical band gap for **1,7-i** (1.56 eV *vs* 1.66 eV), as determined from the onset of absorption of the thin-films at low energy. This result underlines the enhanced π-electronic delocalization of the *1,7*-regioisomer.

**Figure 5:**
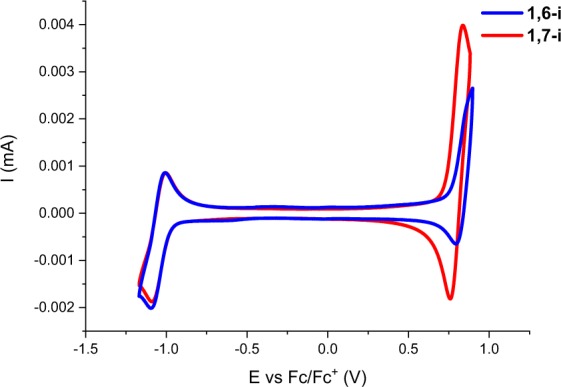
Cyclic voltammograms of **1,7-i** (red) and **1,6-i** (blue) at a concentration of 0.5 mM in 0.10 M Bu_4_NPF_6_/CH_2_Cl_2_, 100 mV s^−1^, Pt working electrode.

The electrochemical properties of the isomers were characterized by cyclic voltammetry, performed in dichloromethane using NBu_4_PF_6_ as supporting electrolyte. One reversible reduction wave at the same reduction peak potential (E_pc_ = −1.05 V *vs* Fc/Fc^+^) was recorded for both regioisomers (Fig. 5).

In the positive potential region, an irreversible oxidation wave was observed at different oxidation peak potentials (E_pa_) of 0.83 V and 0.89 V for **1,7-i** and **1,6-i** respectively, thus revealing a deeper highest occupied molecular orbital (HOMO) level for **1,6-i** (Table 2). The electrochemical gap determined by the difference of the onset of oxidation and reduction was estimated to 1.89 eV and 1.85 eV for **1,6-i** and **1,7-i** respectively (Table 2) following the same trend observed by spectroscopy (Figs. 4 and S19).

**Table 2:** Electrochemical data recorded at a concentration of 0.5 mM in 0.10 M Bu_4_NPF_6_/CH_2_Cl_2_, 100 mV.s^−1^, Pt working electrode, Reference: Fc/Fc^+^.

Compound	E_pa_ (V/_Fc/Fc+_)	E_pc_ (V/_Fc/Fc+_)	E_HOMO_[eV]	E_LUMO_[eV]	ΔE^elec^ [eV]
**1,6-i**	0.89	−1.05	−5.64	−3.75	1.89
**1,7-i**	0.83	−1.05	−5.60	−3.75	1.85

E_HOMO_ = −(E_ox(onset)_ + 4.80) (eV).

E_LUMO_ = −(E_red(onset)_ + 4.80) (eV).

**Figure 6:**
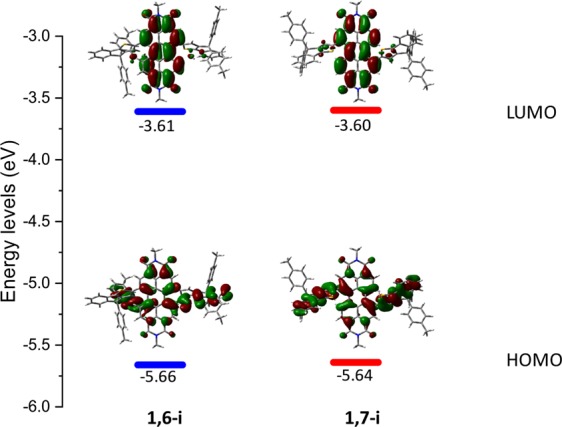
HOMO and LUMO electron density and energy levels calculated by DFT for **1,6-i** and **1,7-i**.

The better conjugation in the *1,7*-substituted derivative and the deeper HOMO level of the *1,6*-regioisomer observed experimentally were afterward investigated from a computational chemistry point of view. Optimized geometries (Fig. S17, Tables S1,S2), orbital density distributions and energies of the frontier molecular levels were simulated by density functional theory (DFT) method using B3LYP model with a 6–311G(d) basis set (Fig. 6).

While LUMO levels are centered on the PDI units, it turns out that the electron densities of the HOMO levels are partially delocalized along the different constituting building blocks. Interestingly, and contrary to **1,6-i**, the HOMO distribution in **1,7-i** appears to be nearly symmetrical and well balanced between the two constituting naphthyl moieties of the PDI core. The charge transfer (CT) character of the lowest energy absorption bands, assigned to HOMO → LUMO transitions, were confirmed by time-dependent DFT (TD-DFT) calculations (Fig. S18 and Table S3). This method also emphasized, through a population analysis, the higher contribution of the IDT arms to the HOMO in **1,7-i** (77% *vs* 71% for **1,6-i**). Hence, with almost similar LUMO levels (88% on the PDI for both isomers), the stronger CT character of **1,7-i** contributes in increasing its HOMO level and therefore reducing its bandgap.

**Figure 7:**
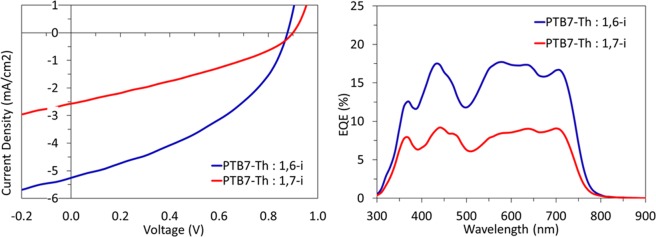
J –V curves (left) and EQE spectra (right) of **1,7-i** (red) and **1,6-i** (blue) based OSCs.

To further investigate the impact of the grafting position of PDI derivatives and more specifically, the impact on their photovoltaic properties, air-processed bulk heterojunction (BHJ) solar cells with inverted architecture (ITO/ZnO/**PTB7-Th**: **1,6-i** or **1,7-i**/MoO_x_/Ag) were fabricated. For the sake of comparison, each regioisomer was first blended with the same donor polymer, namely the **PTB7-Th**, in a 1:1 w/w ratio at 10 mg/mL total concentration in chlorobenzene. The resulting devices were tested and the typical density-voltage (J-V) curves recorded under an AM 1.5 simulated solar illumination in air are depicted in Fig. 7 together with the respective EQE spectra.

Devices prepared with **1,6-i** led to better power conversion efficiencies than those of **1,7-i**, mainly due to the higher short circuit current density (*J*_*sc*_) and fill factor (*FF*) parameters (Table 3). While comparable open circuit voltage (*V*_*oc*_) of 0.86 V for **1,6-i** and 0.89 V for **1,7-i** were measured, in agreement with the similar LUMO levels of the regioisomers, **1,6-i** based devices exhibited a *FF* of 41% and a *J*_*sc*_ of 4.7 mA cm^−2^ *vs* 33% and 2.4 mA cm^−2^ for **1,7-i**, resulting in PCEs of 1.6% *vs* 0.7% respectively. This two-fold reduction of *J*_*sc*_ can also be observed in the external quantum efficiency spectra since the maximum photon-to-electron conversion of **1,7-i** based organic solar cell (OSC) barely exceeded 9% whereas approximatively 18% was reached for **1,6-i** based devices.

**Table 3:** Organic solar cell device parameters for 1:1 PTB7-Th: **1,7-i** and PTB7-Th: **1,6-i** active layers, obtained from 10 mg/mL solutions in chlorobenzene. Average values in brackets.

Acceptor	*V*_*oc*_ (V)		*J*_*sc*_ (mA/cm^2^)		*FF* %		PCE %	
**1,6-i**	0.87	(0.86)	5.3	(4.7)	41	(41)	1.9	(1.6)
**1,7-i**	0.89	(0.89)	2.6	(2.4)	34	(33)	0.8	(0.7)

**Figure 8:**
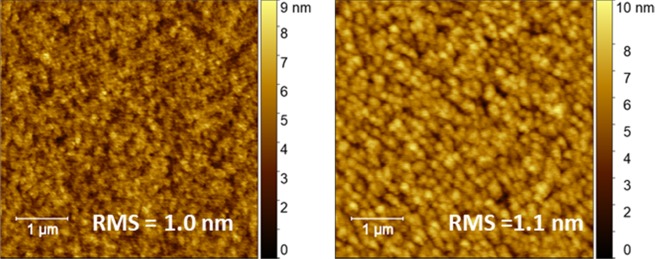
AFM images of **1,6-i** (left) and **1,7-i** (right) based photoactive layers.

The nanoscale topography of the active layers was investigated by atomic force microscopy (AFM). As shown in Fig. 8, both **1,6-i** and **1,7-i** based blends appear similar in roughness, with comparable RMS values of 1.0 and 1.1 nm, respectively.

**Figure 9:**
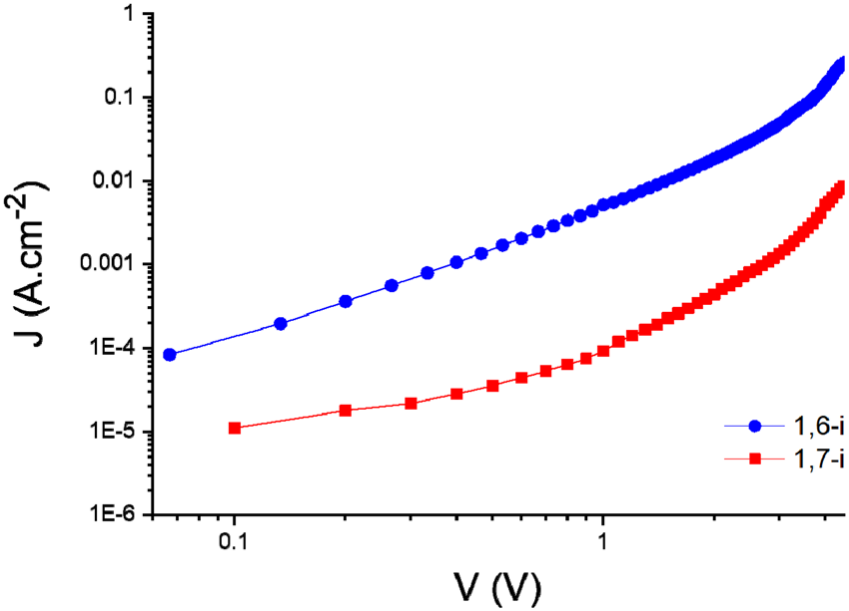
Electron mobility μ_e_ of **1,6-i** (blue) and **1,7-i** (red) based devices.

The slightly more granular nature of the PTB7-Th: **1,7-i** films might be contributing to the lower density of carriers generated (and collected) in the devices, although morphology is hardly the only cause of these low currents and fill factors. Consequently, the electron mobility (μ_e_) of each regioisomer was evaluated and compared using the space charge limited current (SCLC) method (Fig. 9).

**Figure 10:**
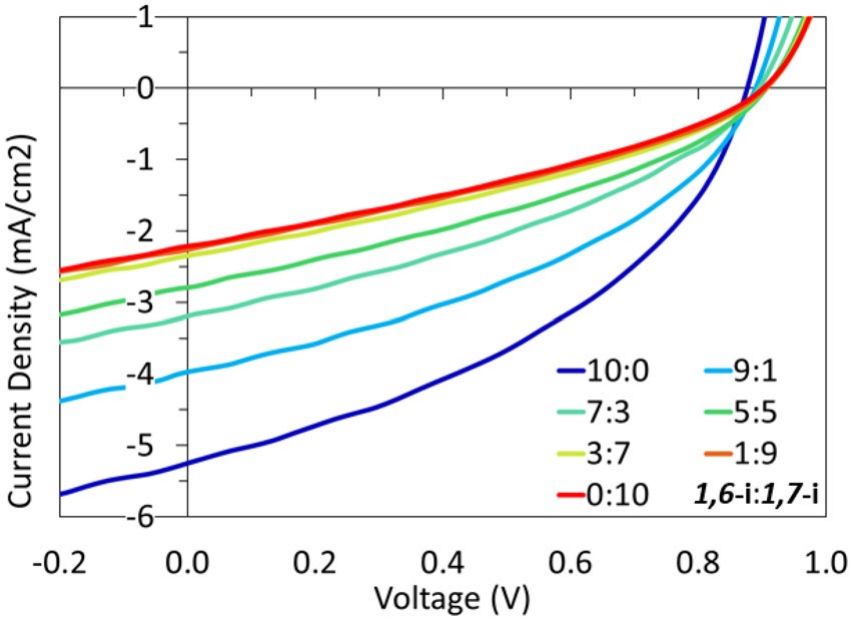
J –V curves of devices prepared with different ratios of **1,6-i** and **1,7-i**.

Electron-only devices (ITO/ZnO/**1,6-i** or **1,7-i**/Al) were thus fabricated revealing a difference of mobility between the two PDI derivatives of *ca* an order of magnitude. While a μ_e_ of *ca* 7.1×10^–6^ cm^2^ V^−1^ s^−1^ was measured for **1,7-i**, the *1,6*-isomer was indeed characterized by an electron mobility of *ca* 8.5×10^–5^ cm^2^ V^−1^ s^−1^, which in consistency with the difference of *Jsc* and *FF*. Consequently, adding an increasing quantity of the *1,7*-isomer to pure **1,6-i** based devices would normally have an impact on their photovoltaic performances, and conversely. Hence, devices made from mixtures of isomers with ratios of 9:1, 7:3, 5:5, 3:7 and 1:9 were finally prepared, characterized and compared to their pure counterparts. As depicted in Fig. 10, increasing the quantity of **1,7-i** results in a significant decrease of both the *Jsc* and *FF* (see Table S5), thus demonstrating that the latter has, in this case, a detrimental effect on the overall performances.

## Conclusion

Generated during the dibromination of the PDIs as a minor product and usually considered as an impurity, the *1,6*-dibromo PDI has not attracted much attention, particularly in the field of organic photovoltaics. In addition to the seminal paper reported by Ge *et al*.^34^, we demonstrate herein that using the more conjugated *1,7*-isomer to prepare new non fullerene acceptors is not an absolute rule to efficiency. In our case, the less conjugated *1,6* derivative indeed led to a two-fold improvement of the power conversion efficiency in comparative devices, which could be slightly enhanced by optimizing the active layer preparation and deposition (PCE up to 1.9%). Moreover, adding an increasing quantity of the *1,7*-isomers to **1,6-i** based devices resulted in a significant decrease of their photovoltaic performances, confirming that the commonly used *1,7-*isomer can be herein considered as a contaminant.

Consequently, this paper highlights the impact of both the grafting position of the PDI and the nature of the end-capping moieties on photovoltaic and charge transport properties and will hopefully contribute in reconsidering conventional design principles and above all, the use of *1,6-*difunctionalized isomer in some organic electronics.

## Materials and Methods

### General information

All reagents and chemicals from commercial sources were used without further purification unless specified. Solvents were dried and purified using standard techniques. Flash chromatography was performed with analytical-grade solvents using ALDRICH silica gel (technical grade, pore size 60 Å, 230–400 mesh particle size). Flexible plates ALUGRAM Xtra SIL G UV254 from MACHEREY-NAGEL were used for TLC. Compounds were detected by UV irradiation (BIOBLOCK SCIENTIFIC). NMR spectra were recorded with a BRUKER AVANCE III 300 (1H, 300 MHz and 13C, 75 MHz) or a BRUKER AVANCE DRX500 (1H, 500 MHz; 13C, 125 MHz). ^13^C APT show CH_2_ and quaternary as positive while CH and CH_3_ as negative. Chemical shifts are given in ppm relative to TMS and coupling constants J in Hz. IR spectra were recorded on a BRUKER spectrometer VERTEX 70 and UV-vis spectra with a PERKIN ELMER 950 spectrometer. Matrix Assisted Laser Desorption/Ionization was performed on MALDI-TOF MS BIFLEX III BRUKER DALTONICS spectrometer using DCTB+ as matrix. High resolution mass spectrometry (HRMS) was performed with a JEOL JMS-700 B/E^38,39^.

Cyclic voltammetry was performed using a BIOLOGIC SP-150 potentiostat with positive feedback compensation in 0.10 M Bu_4_NPF_6_/CH_2_Cl_2_ (HPLC grade). Experiments were carried out in a one-compartment cell equipped with a platinum working electrode (2 mm of diameter) and a platinum wire counter electrode. A silver wire immersed in 0.10 M Bu_4_NPF_6_/CH_2_Cl_2_ was used as pseudo-reference electrode and checked against the ferrocene/ferrocenium couple (Fc/Fc+) before and after each experiment. The potentials were then expressed *vs* Fc/Fc+. X-ray single-crystal diffraction data (Table S4) were collected on an AGILENT SUPERNOVA diffractometer equipped with Atlas CCD detector and micro-focus Cu-K_α_ radiation. The structures were solved by direct methods and refined on F^2^ by full matrix least-squares techniques using SHELX programs (G. M. SHELDRICK 2013–2016). All non-H atoms were refined anisotropically and multiscan empirical absorption was corrected using CRYSALISPRO program (CRYSALISPRO, AGILENT TECHNOLOGIES, 2015–2019). The H atoms were included in the calculation without refinement^40,41^.

The identity of new compounds was confirmed by NMR, IR and high-resolution MALDI-TOF mass spectroscopy (Figs. S1–S16). The synthesis of **(4,4-di-*p*-tolyl-4*H*-indeno[1,2-*b*]thiophen-2-yl)trimethylstannane (4)** was adapted from a preparation reported in the literature^42^. Both **1,6-i** and **1,7-i** isomers were prepared according to a preparation published by Rybtchinski & *al*.^37^.

### Synthesis

#### Methyl 2-(thiophen-2-yl)benzoate (2)

Degassed toluene (30 mL) was added to a mixture of methyl-2-bromobenzoate **1** (5 g, 22.79 mmol) and tributyl(thiophen-2-yl)stannane (10.2 g, 27.34 mmol). Pd(PPh_3_)_4_ (526 mg, 0.46 mmol) was further added before refluxing the reaction mixture for 24 h. The latter was then cooled to room temperature and the solvent removed under *vacuum*. Purification of the crude was performed by column chromatography on silica gel (eluent: petroleum ether/dichloromethane, 1:3) affording 4.97 g of a colourless oil (quantitative). ^**1**^**H-NMR** (300 MHz, CDCl_3_): *δ* 7.73 (dt, *J* = 7.6 Hz, 1.1 Hz, 1H), 7.51–7.47 (m, 2H), 7.40 (ddd, *J* = 8.8 Hz, 7.8 Hz, 4.2 Hz, 1H), 7.35 (dd, *J* = 4.9 Hz, 1.4 Hz, 1H), 7.09–7.02 (m, 2H), 3.78 (s, 3H).

#### 4,4-di-*p***-tolyl-4***H*-indeno[1,2-**b**]thiophene (3)

To a solution of 4-bromotoluene (9.4 g, 54.98 mmol) in distilled THF (40 mL) was added dropwise *n*-BuLi (2.5 M in hexane, 22 mL, 54.98 mmol) at −78 °C. After 1 h, a diluted solution of **2** (4.8 g, 22 mmol) in dry THF (40 mL) was slowly added at −78 °C. The mixture was then stirred for 16 h at room temperature before being poured in water (125 mL). The aqueous layer was extracted with dichloromethane (200 mL × 2), the organic phase was dry over MgSO_4_ and the solvent removed under reduced pressure. The resulting crude was thereafter suspended in a mixture of glacial acetic acid (125 mL) and H_2_SO_4_ (2 mL). After 3 h of reflux, the reaction was cooled to room temperature and the precipitated filtrated and washed with water, ethanol and petroleum ether. Once dried, 4.8 g of a white powder were recovered (62%). ^**1**^**H-NMR** (300 MHz, CDCl_3_): *δ* 7.45 (d, *J* = 7.5 Hz, 1H), 7.35 (d, *J* = 7.6 Hz, 1H), 7.32–7.26 (m, 2H), 7.16 (dt, *J* = 7.5 Hz, 1.1 Hz, 1H), 7.10 (d, *J* = 8.1 Hz, 4H), 7.05–6.99 (m, 1H), 2.29 (s, 6H).

#### (4,4-di-*p*-tolyl-4*H*-indeno[1,2-*b*]thiophen-2-yl)trimethylstannane (4)

**3** (2 g, 5.67 mmol) was dissolved in distilled THF (20 mL) under argon, then *n*-BuLi (2.5 M in hexane, 2.2 mL, 7.94 mmol) was added dropwise at −78 °C and the reaction was stirred for 1 h 30 min. Trimethyl tin chloride (1 M in hexane, 8.5 mL, 8.51 mmol) was then added before warming the reaction mixture to room temperature. The overnight stirred solution was quenched with water and extracted with AcOEt. The organic layer was washed with KF solution (sat.) and water, dry over Mg_2_SO_4_ and the solvent evaporated under *vacuum*. The resulting crude was used without further purification. ^**1**^**H-NMR** (300 MHz, CDCl_3_): *δ* 7.43 (d, *J* = 7.4 Hz, 1H), 7.32 (d, *J* = 7.6 Hz, 1H), 7.26 (td, *J* = 7.6 Hz, *J* = 1,1 Hz, 1H), 7.17–7.07 (m, 5H), 7.08–6.99 (m, 5H), 2.29 (s, 6H), 0.36 (s, 9H).^**13**^**C-NMR** (75 MHz, CDCl_3_): *δ* 157.6, 153.9, 147.1, 142.2, 141.9, 137.3, 136.2, 130.4, 130.0, 127.9, 127.8, 127.3, 126.4, 125.4, 119.5, 62.3, 21.0. **MS** (MALDI-dit+) m/z: 516.0 [M+].

#### Synthesis of 1,6-i and 1,7-i

To a blend of halogenated perylene diimide (**1,7-** and **1,6-PDI**) (150 mg, 0.25 mmol) were added **4** (317 mg, 0.62 mmol), Pd(PPh_3_)_4_ (25 mg, 0.02 mmol) and 10 ml of dry toluene. The reaction mixture was then stirred and refluxed for 24 h under inert conditions. Once concentrated under *vacuum*, the crude was purified by column chromatography (eluent: dichloromethane/petroleum ether) affording **(1,6-i)** and **(1,7-i)** in 21% and 79% yield respectively. **(1,6-i)**. ^**1**^**H-NMR** (300 MHz, CDCl_3_): *δ* 8.64 (s, 2H), 8.38 (d, *J* = 8.1 Hz, 2H), 8.26 (d, *J* = 8.2 Hz, 2H), 7.53 (d, *J* = 7.6 Hz, 2H), 7.42–7.32 (m, 4H), 7.24 (td, *J* = 7.6, 1.2 Hz, 2H), 7.10–7.00 (m, 16H), 6.96 (s, 2H), 5.16–4.97 (m, 2H), 2.42–1.78 (m, 20H), 0.94 (t, *J* = 7.6 Hz, 12H).^**13**^**C-NMR** (75 MHz, CDCl_3_): *δ* 157.5, 153.5, 146.5, 143.5, 141.3, 136.9, 136.8, 134.9, 134.0, 129.6, 129.4, 129.2, 129.0, 128.9, 128.0, 127.9, 127.8, 126.6, 126.4, 123.1, 122.4, 120.0, 63.5, 57.9, 57.7, 25.2, 25.1, 21.1, 11.6, 11.4. **IR** (neat): *ν* = 3090–3015 cm^−1^ (C_sp2_-H, Ar), 2965–2862 cm^−1^ (C_sp3_-H), 1699 cm^−1^ (C=O), 1653 cm^−1^ (C=O), 1580–1458 cm^−1^ (C=C, Ar), 1336 cm^−1^ (C_sp3_-N), 1321 cm^−1^ (C_sp3_-N). **UV-Vis** (CH_2_Cl_2_): λ_max_ (ε) = 573 nm (16000 L.mol^−1^.cm^−1^), 456 nm (17000 L.mol^−1^.cm^−1^), 316 nm (41000 L.mol^−1^.cm^−1^). **MS** (MALDI-dctb+) m/z: 1230.6 [M+]. **HRMS** (FAB+): calculated for C_84_H_66_N_2_O_4_S_2_ 1230.4458, found 1230.4446. **(1,7-i)** was obtained in the second fraction as a green solid. (240 mg, 79%). ^**1**^**H-NMR** (300 MHz, CDCl_3_): *δ* 8.67 (s, 2H), 8.38 (d, *J* = 8.2 Hz, 2H), 8.22 (d, *J* = 8.2 Hz, 2H), 7.53 (d, *J* = 7.3 Hz, 2H), 7.42–7.32 (m, 4H), 7.25 (td, *J* = 7.5, 1.1 Hz, 2H), 7.17 (s, 2H), 7.08 (d, *J* = 8.5 Hz, 8H), 7.03 (d, *J* = 8.2 Hz, 8H), 5.11–5.00 (m, 2H), 2.35–2.18 (m, 16H), 2.00–1.84 (m, 4H), 0.91 (t, *J* = 7.5 Hz, 12H).^**13**^**C-NMR** (75 MHz, CDCl_3_): *δ* 157.7, 153.5, 146.3, 143.8, 141.3, 136.8, 136.8, 135.0, 133.8, 129.8, 129.5, 129.2, 128.4, 127.9, 127.8, 126.7, 126.5,123.8, 120.1, 63.5, 57.8, 25.2, 21.1, 11.5. **IR** (neat): *ν* = 3056–3018 cm^−1^ (C_sp2_-H, Ar), 2962–2876 cm^−1^ (C_sp3_-H), 1696 cm^−1^ (C=O), 1654 cm^−1^ (C=O), 1594–1506 cm^−1^ (C=C, Ar), 1324 cm^−1^ (C_sp3_-N). **UV-Vis** (CH_2_Cl_2_): λ_max_ (ε) = 630 nm (14000 L.mol^−1^.cm^−1^), 465 nm (17000 L.mol^−1^.cm^−1^), 336 nm (41000 L.mol^−1^.cm^−1^). **MS** (MALDI-dctb+) m/z: 1230.6 [M+]. **HRMS** (FAB+): calculated for C_84_H_66_N_2_O_4_S_2_ 1230.4458, found 1230.4434.

#### Computational calculation methodology

Ground-state density-functional theory (DFT) geometry optimizations and time-dependent density-functional theory (TD-DFT) calculations were carried out using GAUSSIAN 16^43^ suite package. B3LYP/6–311G* basis sets were chosen for all the atomic species. In order to take into account solvation effects in the reproduction of the absorption spectra, solvent dichloromethane molecules were treated as a polarizable continuum (PCM).

#### Organic photovoltaic devices fabrication and characterization

PTB7-Th: **1,6-i** or **1,7-i** solutions were prepared in air, from 10 mg/mL solutions of the single components, which were stirred for approximatively 2 hours before mixing in the required proportions. The final solutions were stirred for at least 1 hour before deposition on substrates. ZnO precursor solutions were prepared following the sol-gel method proposed by Sun *et* *al*.^44^, 1.0 g of zinc acetate dihydrate, 0.280 mL of ethanolamine and 10.0 mL of 2-methoxy ethanol were mixed in air and stirred overnight at room temperature before use. All studied films were prepared as follow: ITO-coated glass substrates were first cleaned by surfactant/water scrubbing, followed by sequentially ultra-sonicating in de-ionized water, acetone and isopropanol (10+ minutes each) before use. ITO substrates were then dried with pressurized air and UV-Ozone treated for 30 minutes. A ZnO precursor solution was spin-coated onto the ITO substrate at a speed of 4200 rpm for 55 s and then thermally annealed at 200 °C in air for 20 min. The organic layer was then spin-coated at room temperature, in air at 1000 rpm for 50 s. Solar cells were fabricated following the initial procedure for cleaning, ZnO deposition and organic layer deposition reported above. The fabricated films were then moved to an N_2_ atmosphere glovebox and left overnight before evaporating the top electrodes consisting of 10 nm of MoO_x_ (electron transport interlayer) followed by 100 nm of Ag (anode), which were thermally deposited under high vacuum (10^–5^ mbar). Current density-voltage (J-V) characteristics were measured using a Keithley 2420 Source Measure Unit. Solar cell performance used an Air Mass 1.5 Global (AM 1.5G) Solar Simulator (Newport, Model 92251A-1000) with an irradiation intensity of 100 mWcm-2, which was measured by a calibrated silicon solar cell and a readout meter (Newport, Model 91150V).

#### Atomic force microscopy (AFM)

AFM measurements were performed by using a TT-2 AFM (AFM Workshop, USA) in the tapping mode and WSxM software with a 0.01–0.025 Ohm/cm Sb (n) doped Si probe with a reflective back side aluminium coating.

#### SCLC measurements

A solution of neat isomers, namely **1,6-i** or **1,7-i** (20 mg/mL) in chloroform was spun-cast at 1000 rpm on the above described ZnO coated substrates to provide organic layers of *ca*. 140 nm and 180 nm respectively. Calcium (7 nm) and aluminium (100 nm) were thermally evaporated under a *vacuum* of 1.5 × 10^−5^ Torr, through a shadow mask defining active areas of 12.60 mm², 3.10 mm² and 0.78 mm² per substrate. Electron mobilities μ_e_ were evaluated using the Mott-Gurney law, ie, JSCLC = (9/8)ε_0_ε_r_μ_e_(V^2^/d^3^) where εr is the static dielectric constant of the medium (ε_r_ = 3) and d, the thickness of the active layer.

## Supplementary information

Supplementary information.
