# Supplementary material for: Indeno[1,2-b]thiophene End-capped Perylene Diimide: Should the 1,6-Regioisomers be systematically considered as a byproduct?
Source: Sci Rep. 2020 Feb 24;10:3262. doi: 10.1038/s41598-020-60012-7 (PMC7039957; doi:10.1038/s41598-020-60012-7)
Supplement: Supplementary file 1 — Supplementary information. [file 41598_2020_60012_MOESM1_ESM.docx]

**Supporting Information**

**Indeno[1,2-*b*]thiophene End-capped Perylene Diimide: Should the *1,6*- Regioisomers be systematically considered as a byproduct?**

Pablo Simón Marqués,^1^ Francesco Tintori,^2^ José María Andrés Castán,^1^ Pierre Josse,^1^ Clément Dalinot,^1^ Magali Allain, ^1^ Gregory Welch,^2^ Philippe Blanchard^1^ and Clément Cabanetos^1^

^1^CNRS UMR 6200, MOLTECH-Anjou, University of Angers, 2 Bd Lavoisier, 49045 Angers, France

^2^Department of Chemistry, University of Calgary, 2500 University Drive N.W., Calgary, Alberta T2N 1N4, Canada

**Table of contents**

**^1^H, ^13^C, 2D NMR and IR Spectra**

**Mass Spectrometry**

**DFT Calculation**

**Photoelectron Spectroscopy in Air (PESA)**

**Crystal Structure Data**

**Photovoltaic data**

**^1^H, ^13^C NMR and IR spectra**

 **Figure S1.** ^1^H NMR (CDCl_3_, 300 MHz) spectrum of **2**.

**2**

 **Figure S2.** ^1^H NMR (CDCl_3_, 300 MHz) spectrum of **3**.

**3**

 **Figure S3.** ^1^H NMR (CDCl_3_, 300 MHz) spectrum of **4** **Figure S4.** ^13^C NMR (CDCl_3_, 75 MHz) spectrum of **4**.

**4**

**** **Figure S5.** ^1^H NMR (CDCl_3_, 300 MHz) spectrum of **1,6-i**.

**1,6-i**

 **Figure S6.** ^13^C APT (CDCl_3_, 75 MHz) spectrum of **1,6-i**.

**1,6-i**

 **Figure S7.** 2D COSY (CDCl_3_, 300 MHz) spectrum of **1,6-i**.

**1,6-i**

 **Figure S8.** ^1^H NMR (CDCl_3_, 300 MHz) spectrum of **1,7-i**.

**1,7-i**

 **Figure S9.** ^13^C APT (CDCl_3_, 75 MHz) spectrum of **1,7-i.**

**1,7-i**

 **Figure S10.** ^1^H NMR (CDCl_3_, 300 MHz). Aromatic zoom of **1,6-i** (blue) and **1,7-i** (red).

**1,7-i**

**1,6-i**

**1,7-i**

**1,6-i**

**Figure S11.** ^13^C APT (CDCl_3_, 75 MHz). Aliphatic zoom of **1,6-i** (blue) and **1,7-i** (red).

**1,6-i**

**1,7-i**

**Figure S12.** IR (Neat product) spectra of **1,6-i** and **1,7-i**.

**Mass Spectrometry**


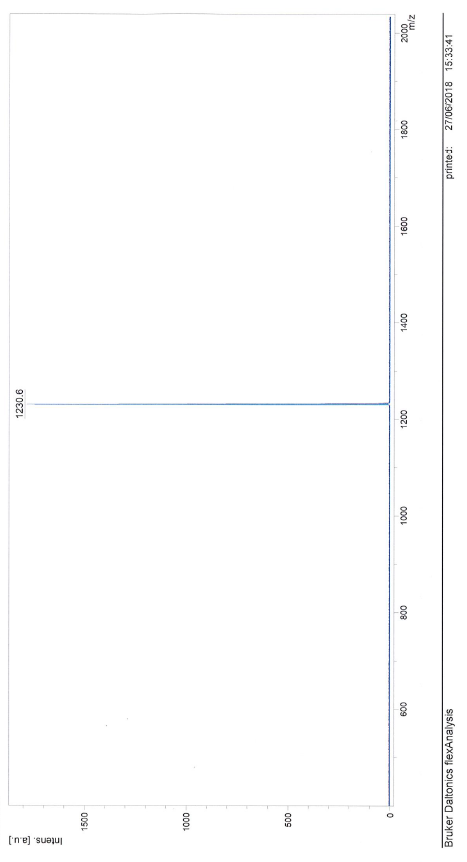
**Figure S13.** MS (MALDI-dctb+) of **1,6-i**.

**1,6-i**


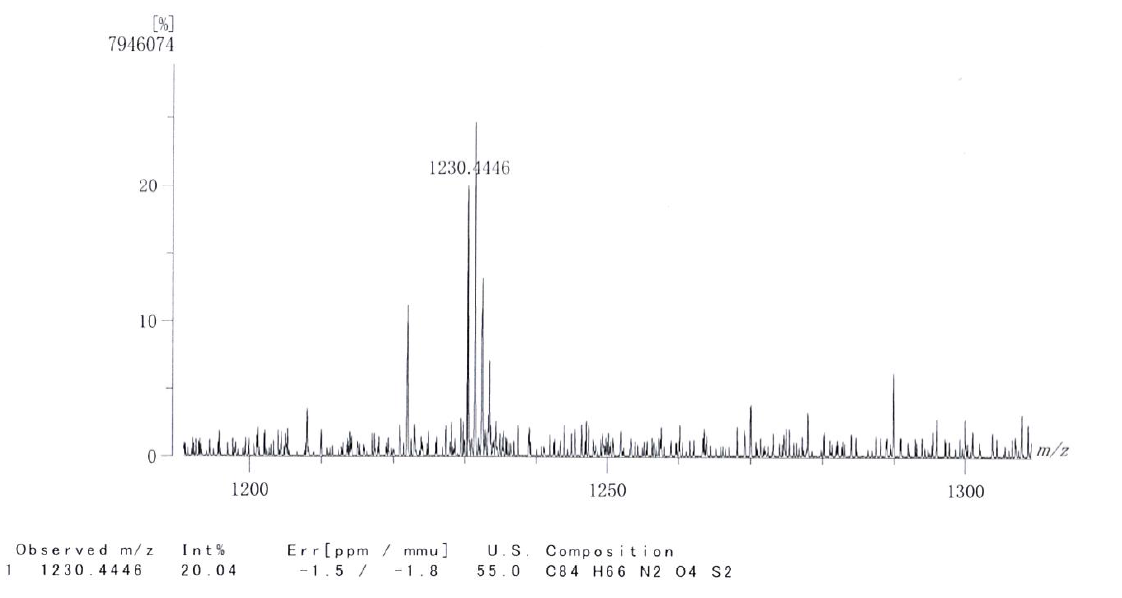
**Figure S14.** HRMS (FAB+) of **1,6-i**. Calculated for C_84_H_66_N_2_O_4_S_2_ 1230.4458, found 1230.4446.

**1,6-i**


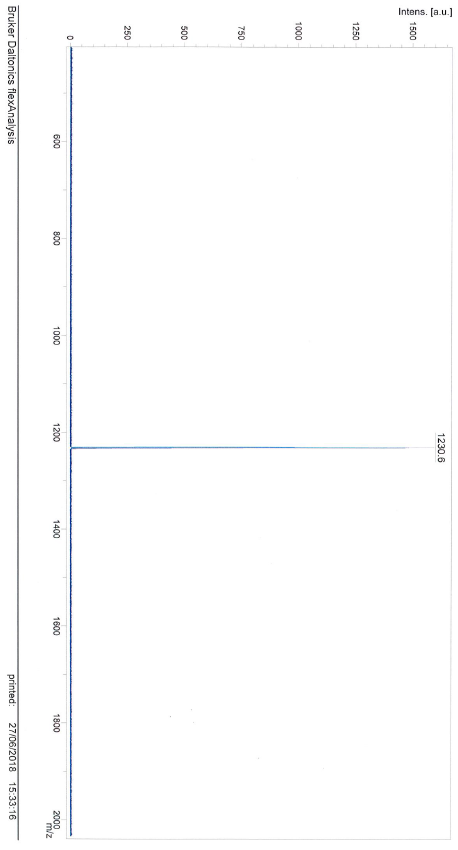
**Figure S15.** MS (MALDI-dctb+) of **1,7-i**.

**1,7-i**


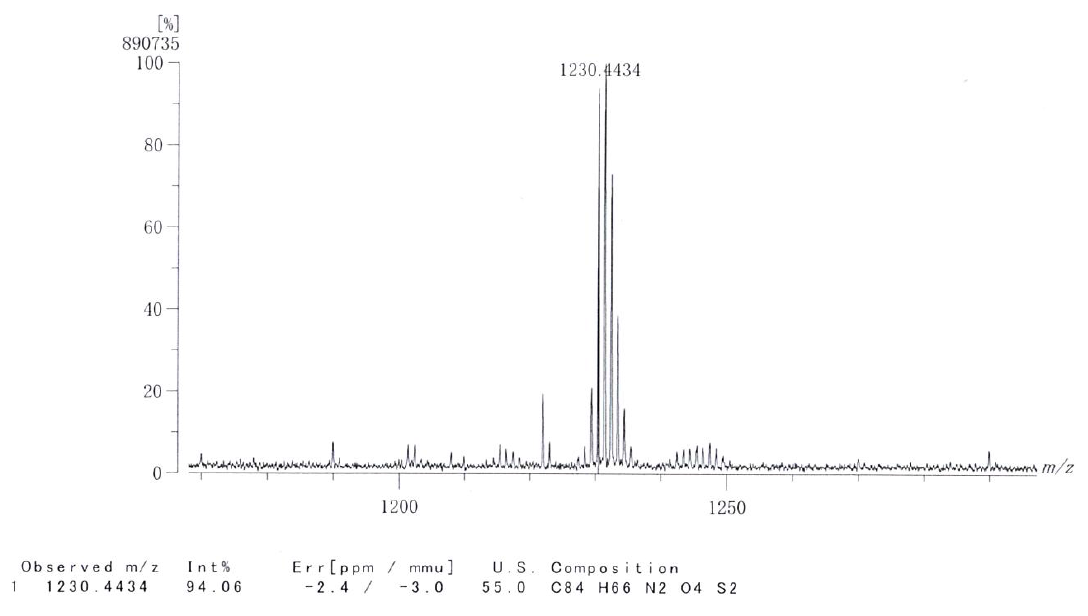
**Figure S16.** HRMS (FAB+) of **1,7-i**. Calculated for C_84_H_66_N_2_O_4_S_2_ 1230.4458, found 1230.4434.

**1,7-i**

**DFT Calculation**

**Table S1.** Optimized Z-Matrixes of **1,7-i** and **1,6-i** at B3LYP/6-311G(d) in DCM.

| **1,7-i** | | | | | **1,6-i** | | | | |
| --- | --- | --- | --- | --- | --- | --- | --- | --- | --- |
| Tag | Symbol | X | Y | Z | Tag | Symbol | X | Y | Z |
| 1 | C | -1.1684988 | 0.8223084 | 0.7477936 | 1 | C | -2.8858688 | 2.5662148 | -2.1864469 |
| 2 | C | -1.3079112 | -0.6205997 | 0.5027053 | 2 | C | -2.7434697 | 1.182684 | -2.1043284 |
| 3 | C | -0.1324893 | -1.4152233 | 0.6846925 | 3 | C | -1.565839 | 0.5812777 | -1.6539478 |
| 4 | C | 1.168499 | -0.8223067 | 0.7477953 | 4 | C | -0.4347838 | 1.4181906 | -1.4025379 |
| 5 | C | 1.3079117 | 0.6206011 | 0.5027062 | 5 | C | -0.5875974 | 2.8376195 | -1.5036046 |
| 6 | C | 0.1324897 | 1.4152248 | 0.6846919 | 6 | C | -1.8363226 | 3.3997007 | -1.8504242 |
| 7 | C | -0.2503854 | -2.8284815 | 0.8167479 | 7 | C | 0.8613478 | 0.8734573 | -1.133817 |
| 8 | C | 0.8783135 | -3.6150501 | 1.1310725 | 8 | C | 1.937082 | 1.76001 | -1.047391 |
| 9 | C | 2.1074499 | -3.0048724 | 1.3043634 | 9 | C | 1.7720153 | 3.1433494 | -1.1132163 |
| 10 | C | 2.2498567 | -1.6315587 | 1.0990204 | 10 | C | 0.5216007 | 3.6933321 | -1.314 |
| 11 | C | -2.5230552 | -1.2685217 | 0.1903729 | 11 | C | -1.4129575 | -0.8782799 | -1.5713809 |
| 12 | C | -2.597656 | -2.6748681 | 0.2882619 | 12 | C | -0.082736 | -1.4126313 | -1.470921 |
| 13 | C | -1.5104698 | -3.442881 | 0.6410554 | 13 | C | 1.0361338 | -0.5884268 | -1.0988963 |
| 14 | C | 0.2503859 | 2.8284831 | 0.816747 | 14 | C | -2.4997055 | -1.7860913 | -1.6115127 |
| 15 | C | -0.8783133 | 3.615052 | 1.1310698 | 15 | C | -2.2556285 | -3.1466652 | -1.8827744 |
| 16 | C | -2.10745 | 3.0048745 | 1.3043592 | 16 | C | -0.978305 | -3.6505214 | -1.9612984 |
| 17 | C | -2.2498568 | 1.6315608 | 1.0990169 | 17 | C | 0.1155378 | -2.8097165 | -1.6698883 |
| 18 | C | 2.5230558 | 1.2685225 | 0.1903739 | 18 | C | 1.4012938 | -3.3731743 | -1.5392133 |
| 19 | C | 2.5976571 | 2.6748689 | 0.2882629 | 19 | C | 2.4243977 | -2.5980672 | -1.0448528 |
| 20 | C | 1.5104708 | 3.4428821 | 0.6410557 | 20 | C | 2.2483599 | -1.2319232 | -0.7463408 |
| 21 | C | -1.6700617 | -4.9098852 | 0.7771418 | 21 | C | 0.3785016 | 5.1654288 | -1.3811239 |
| 22 | N | -0.5199285 | -5.6348603 | 1.1120932 | 22 | N | -0.9003558 | 5.6606111 | -1.6663074 |
| 23 | C | 0.7513288 | -5.0786634 | 1.2967597 | 23 | C | -2.0216825 | 4.8644974 | -1.9107081 |
| 24 | C | 1.670063 | 4.9098863 | 0.7771424 | 24 | C | -0.777894 | -5.0889181 | -2.2470203 |
| 25 | N | 0.5199294 | 5.6348619 | 1.1120928 | 25 | N | 0.5323175 | -5.5717284 | -2.1888698 |
| 26 | C | -0.7513288 | 5.0786654 | 1.2967568 | 26 | C | 1.6475178 | -4.8059074 | -1.8272926 |
| 27 | O | 2.7432466 | 5.4670952 | 0.6107838 | 27 | O | 1.3206417 | 5.9198325 | -1.1966134 |
| 28 | O | -1.696105 | 5.796468 | 1.583661 | 28 | O | -3.0999863 | 5.3843796 | -2.1580645 |
| 29 | O | 1.6961048 | -5.7964656 | 1.5836667 | 29 | O | 2.7602715 | -5.3012368 | -1.7589087 |
| 30 | O | -2.743245 | -5.4670944 | 0.610782 | 30 | O | -1.6975318 | -5.8401748 | -2.5282567 |
| 31 | C | -0.6258665 | -7.0897777 | 1.2770225 | 31 | C | -1.1005095 | 7.1134366 | -1.7265108 |
| 32 | C | 0.6258681 | 7.0897791 | 1.2770236 | 32 | C | 0.7209159 | -6.9949055 | -2.4957529 |
| 33 | C | -8.6914493 | 2.5298426 | -3.4779461 | 33 | C | 3.3599033 | -0.6250727 | 0.0129817 |
| 34 | C | -7.4607074 | 2.7468587 | -4.0998248 | 34 | C | 4.6989755 | -0.6112679 | -0.3036312 |
| 35 | C | -6.2943812 | 2.1804511 | -3.5840614 | 35 | C | 5.4975218 | -0.0472088 | 0.7232107 |
| 36 | C | -6.3842015 | 1.398644 | -2.4334204 | 36 | C | 4.7709537 | 0.3383368 | 1.8221039 |
| 37 | C | -7.6273027 | 1.1800179 | -1.7973198 | 37 | S | 3.0796786 | 0.0205518 | 1.6349692 |
| 38 | C | -8.7798918 | 1.7405667 | -2.325057 | 38 | C | 6.9937133 | 0.1855652 | 0.9529756 |
| 39 | C | -7.4696252 | 0.2667084 | -0.558697 | 39 | C | 6.9556208 | 0.7953312 | 2.3743377 |
| 40 | C | -5.3887691 | 0.6709329 | -1.6654438 | 40 | C | 5.631838 | 0.8742556 | 2.8625515 |
| 41 | S | -3.6775719 | 0.4283092 | -1.7410451 | 41 | C | 8.0058879 | 1.2624802 | 3.1486685 |
| 42 | C | -3.7369839 | -0.5922678 | -0.2998863 | 42 | C | 7.7382918 | 1.792927 | 4.416347 |
| 43 | C | -5.0273128 | -0.7289815 | 0.1604384 | 43 | C | 6.4297088 | 1.8619597 | 4.8975432 |
| 44 | C | -5.9630255 | 0.0011221 | -0.6136052 | 44 | C | 5.3625251 | 1.4054485 | 4.122885 |
| 45 | C | -8.3202653 | -1.0021229 | -0.7677501 | 45 | C | 7.6128482 | 1.2201319 | -0.0083333 |
| 46 | C | -7.7851695 | 0.97544 | 0.7754068 | 46 | C | 7.7016817 | -1.1844715 | 0.8894009 |
| 47 | C | -7.9014939 | 2.3628232 | 0.8794503 | 47 | C | 7.9886754 | -1.9326499 | 2.0330478 |
| 48 | C | -8.1079606 | 2.9768072 | 2.116343 | 48 | C | 8.5540767 | -3.2049969 | 1.9373399 |
| 49 | C | -8.2049382 | 2.2315396 | 3.2920178 | 49 | C | 8.8521244 | -3.7784941 | 0.7002076 |
| 50 | C | -8.0827597 | 0.8396202 | 3.1864011 | 50 | C | 8.5561022 | -3.030144 | -0.4461827 |
| 51 | C | -7.8772759 | 0.2248942 | 1.9570522 | 51 | C | 7.9929621 | -1.7622638 | -0.3548697 |
| 52 | C | -9.6727506 | -1.0239477 | -0.4034042 | 52 | C | 8.9962411 | 1.2750282 | -0.2106002 |
| 53 | C | -10.467957 | -2.1370946 | -0.6581945 | 53 | C | 9.5674731 | 2.2580044 | -1.0164842 |
| 54 | C | -9.9470616 | -3.2756319 | -1.2839851 | 54 | C | 8.7827405 | 3.225571 | -1.6502021 |
| 55 | C | -8.599469 | -3.249521 | -1.6509994 | 55 | C | 7.4000861 | 3.1698206 | -1.4454173 |
| 56 | C | -7.8002403 | -2.1349269 | -1.399667 | 56 | C | 6.8262483 | 2.1896615 | -0.6399029 |
| 57 | C | -10.806196 | -4.4923412 | -1.5268173 | 57 | C | 9.4000745 | 4.302658 | -2.5084044 |
| 58 | C | -8.4460743 | 2.8889931 | 4.628846 | 58 | C | 9.4882756 | -5.1428679 | 0.5951849 |
| 59 | C | 8.6914463 | -2.5298468 | -3.4779454 | 59 | C | -3.8889177 | -1.4292806 | -1.2699097 |
| 60 | C | 7.4607041 | -2.7468619 | -4.099824 | 60 | C | -4.3390904 | -0.831522 | -0.1147301 |
| 61 | C | 6.2943785 | -2.1804528 | -3.5840605 | 61 | C | -5.7488064 | -0.7089433 | -0.0773089 |
| 62 | C | 6.3841999 | -1.3986454 | -2.4334198 | 62 | C | -6.3669465 | -1.216492 | -1.1947061 |
| 63 | C | 7.6273013 | -1.1800205 | -1.7973195 | 63 | S | -5.2340883 | -1.8691011 | -2.3279534 |
| 64 | C | 8.7798898 | -1.7405705 | -2.3250566 | 64 | C | -6.7857461 | -0.2094297 | 0.9341576 |
| 65 | C | 7.4696249 | -0.2667096 | -0.5586975 | 65 | C | -8.0873611 | -0.4913863 | 0.146905 |
| 66 | C | 5.3887682 | -0.6709334 | -1.6654435 | 66 | C | -7.8109418 | -1.0875875 | -1.1044424 |
| 67 | S | 3.677571 | -0.4283095 | -1.7410436 | 67 | C | -9.3976565 | -0.2179551 | 0.5062745 |
| 68 | C | 3.736984 | 0.5922681 | -0.299885 | 68 | C | -10.4333688 | -0.5535662 | -0.3738652 |
| 69 | C | 5.0273131 | 0.7289813 | 0.1604388 | 69 | C | -10.15615 | -1.1496554 | -1.6052775 |
| 70 | C | 5.9630254 | -0.0011225 | -0.6136053 | 70 | C | -8.8405828 | -1.4201799 | -1.9837423 |
| 71 | C | 8.3202657 | 1.002121 | -0.767752 | 71 | C | -6.6941462 | 1.3023897 | 1.221236 |
| 72 | C | 7.7851693 | -0.97544 | 0.775407 | 72 | C | -6.6465559 | -1.060449 | 2.2151724 |
| 73 | C | 7.901492 | -2.3628232 | 0.8794522 | 73 | C | -7.4430973 | -2.1800884 | 2.4620899 |
| 74 | C | 8.1079587 | -2.9768059 | 2.1163456 | 74 | C | -7.2362003 | -2.9751204 | 3.5906942 |
| 75 | C | 8.204938 | -2.2315367 | 3.2920194 | 75 | C | -6.228597 | -2.6842353 | 4.5115036 |
| 76 | C | 8.0827606 | -0.8396174 | 3.1864009 | 76 | C | -5.426157 | -1.564022 | 4.258504 |
| 77 | C | 7.8772766 | -0.2248929 | 1.9570513 | 77 | C | -5.6296489 | -0.769615 | 3.1362663 |
| 78 | C | 9.6727517 | 1.0239451 | -0.4034077 | 78 | C | -7.3083454 | 1.8542479 | 2.3506535 |
| 79 | C | 10.4679584 | 2.1370913 | -0.6581989 | 79 | C | -7.2898605 | 3.228692 | 2.5808417 |
| 80 | C | 9.947063 | 3.2756291 | -1.2839884 | 80 | C | -6.657406 | 4.1060665 | 1.6956698 |
| 81 | C | 8.5994699 | 3.2495193 | -1.6510007 | 81 | C | -6.0449596 | 3.5528862 | 0.5659813 |
| 82 | C | 7.8002407 | 2.1349255 | -1.3996676 | 82 | C | -6.0656634 | 2.1805823 | 0.331551 |
| 83 | C | 10.8061981 | 4.4923376 | -1.526822 | 83 | C | -6.6348347 | 5.5952845 | 1.9396147 |
| 84 | C | 8.4460751 | -2.8889889 | 4.6288481 | 84 | C | -6.015795 | -3.5286474 | 5.7439397 |
| 85 | H | 2.9642044 | -3.6069284 | 1.5809691 | 85 | H | -3.819946 | 2.9991502 | -2.5226727 |
| 86 | H | 3.230653 | -1.1952765 | 1.2188037 | 86 | H | -3.5784543 | 0.5721953 | -2.4079038 |
| 87 | H | -3.5366063 | -3.169187 | 0.0726503 | 87 | H | 2.9401992 | 1.3805646 | -0.9434154 |
| 88 | H | -2.9642048 | 3.6069309 | 1.5809635 | 88 | H | 2.6299167 | 3.7980599 | -1.021643 |
| 89 | H | -3.2306533 | 1.1952788 | 1.2187991 | 89 | H | -3.0898092 | -3.8299369 | -1.9835541 |
| 90 | H | 3.5366076 | 3.1691877 | 0.0726519 | 90 | H | 3.3782287 | -3.0640648 | -0.8322578 |
| 91 | H | -0.2627115 | -7.3755221 | 2.263361 | 91 | H | -0.1455804 | 7.5920926 | -1.5410259 |
| 92 | H | 0.2627041 | 7.3755234 | 2.2633586 | 92 | H | -1.8263407 | 7.4201566 | -0.9734355 |
| 93 | H | -9.5901515 | 2.9738349 | -3.8929674 | 93 | H | -1.4779314 | 7.3951175 | -2.7091061 |
| 94 | H | -7.4100621 | 3.3583368 | -4.9946515 | 94 | H | 1.7837389 | -7.2071242 | -2.4670968 |
| 95 | H | -5.3396784 | 2.3448536 | -4.0727939 | 95 | H | 0.3179812 | -7.2139166 | -3.4836557 |
| 96 | H | -9.7442013 | 1.5743941 | -1.8568021 | 96 | H | 0.1982064 | -7.608242 | -1.7614306 |
| 97 | H | -5.2691651 | -1.3158706 | 1.037779 | 97 | H | 5.0721424 | -0.9757935 | -1.252602 |
| 98 | H | -7.8304933 | 2.9816753 | -0.0069969 | 98 | H | 9.0268659 | 1.2195152 | 2.7843981 |
| 99 | H | -8.1953954 | 4.0587776 | 2.1587356 | 99 | H | 8.5570452 | 2.1559293 | 5.0286186 |
| 100 | H | -8.1508687 | 0.2261332 | 4.0807457 | 100 | H | 6.2391254 | 2.2783096 | 5.8811662 |
| 101 | H | -7.7969754 | -0.8561839 | 1.9164645 | 101 | H | 4.3454242 | 1.4672751 | 4.4957228 |
| 102 | H | -10.1121357 | -0.1663459 | 0.0940254 | 102 | H | 7.7700221 | -1.5298877 | 3.0147615 |
| 103 | H | -11.5132343 | -2.1192962 | -0.3619059 | 103 | H | 8.7648772 | -3.7572363 | 2.8488377 |
| 104 | H | -8.1605136 | -4.1134692 | -2.1420503 | 104 | H | 8.7683087 | -3.4457762 | -1.4274442 |
| 105 | H | -6.7606141 | -2.1535956 | -1.7068678 | 105 | H | 7.7851275 | -1.2124804 | -1.2666234 |
| 106 | H | -11.8186023 | -4.21437 | -1.8309987 | 106 | H | 9.6405966 | 0.5359521 | 0.2521956 |
| 107 | H | -10.3843064 | -5.1330099 | -2.3041048 | 107 | H | 10.6448547 | 2.2661247 | -1.1558291 |
| 108 | H | -10.8989955 | -5.0988386 | -0.6192655 | 108 | H | 6.7572096 | 3.9023226 | -1.9258967 |
| 109 | H | -8.3712984 | 3.9760306 | 4.5601757 | 109 | H | 5.7509033 | 2.1853994 | -0.5021683 |
| 110 | H | -9.4423488 | 2.649988 | 5.0151656 | 110 | H | 10.4216639 | 4.0484732 | -2.7984541 |
| 111 | H | -7.7243942 | 2.5496458 | 5.3775229 | 111 | H | 8.8220783 | 4.4694391 | -3.4212284 |
| 112 | H | 9.5901482 | -2.97384 | -3.8929669 | 112 | H | 9.4393952 | 5.2587457 | -1.9753585 |
| 113 | H | 7.4100581 | -3.3583403 | -4.9946505 | 113 | H | 9.3904097 | -5.7034447 | 1.5271336 |
| 114 | H | 5.3396756 | -2.3448547 | -4.0727927 | 114 | H | 10.5574805 | -5.0652858 | 0.370076 |
| 115 | H | 9.7441995 | -1.5743985 | -1.8568022 | 115 | H | 9.0356535 | -5.7357935 | -0.2040183 |
| 116 | H | 5.2691661 | 1.3158705 | 1.0377792 | 116 | H | -3.6642092 | -0.5074586 | 0.6673461 |
| 117 | H | 7.8304898 | -2.9816764 | -0.0069941 | 117 | H | -9.6271276 | 0.2499661 | 1.4576351 |
| 118 | H | 8.1953919 | -4.0587765 | 2.1587396 | 118 | H | -11.4610665 | -0.3453717 | -0.0954584 |
| 119 | H | 8.1508707 | -0.2261294 | 4.0807447 | 119 | H | -10.970154 | -1.4013117 | -2.2770146 |
| 120 | H | 7.7969773 | 0.8561852 | 1.9164623 | 120 | H | -8.6277513 | -1.8768468 | -2.9447618 |
| 121 | H | 10.1121366 | 0.1663428 | 0.0940212 | 121 | H | -8.2346723 | -2.4457825 | 1.7717216 |
| 122 | H | 11.5132361 | 2.1192922 | -0.3619116 | 122 | H | -7.8764324 | -3.8379004 | 3.7517078 |
| 123 | H | 8.1605143 | 4.1134679 | -2.1420506 | 123 | H | -4.629144 | -1.3083366 | 4.9514659 |
| 124 | H | 6.760614 | 2.1535953 | -1.7068669 | 124 | H | -4.9930555 | 0.0946661 | 2.980238 |
| 125 | H | 11.8185932 | 4.2143661 | -1.8310403 | 125 | H | -7.7966626 | 1.2080336 | 3.0717754 |
| 126 | H | 10.3842887 | 5.1330249 | -2.3040835 | 126 | H | -7.7733096 | 3.6214583 | 3.4708958 |
| 127 | H | 10.8990298 | 5.0988157 | -0.6192606 | 127 | H | -5.536518 | 4.2027368 | -0.1413136 |
| 128 | H | 8.371289 | -3.9760259 | 4.560181 | 128 | H | -5.5827756 | 1.7923374 | -0.5574951 |
| 129 | H | 9.4423541 | -2.6499922 | 5.0151614 | 129 | H | -7.0558459 | 5.8483693 | 2.9146977 |
| 130 | H | 7.7244024 | -2.5496329 | 5.3775281 | 130 | H | -5.6158393 | 5.9911487 | 1.9014468 |
| 131 | H | -1.6682246 | -7.3662363 | 1.1651361 | 131 | H | -7.2130894 | 6.1310458 | 1.1799332 |
| 132 | H | -0.0228516 | -7.5963805 | 0.5230952 | 132 | H | -6.568267 | -4.4687064 | 5.6862865 |
| 133 | H | 1.6682277 | 7.3662362 | 1.1651467 | 133 | H | -4.9584936 | -3.7681579 | 5.887476 |
| 134 | H | 0.0228614 | 7.5963833 | 0.5230906 | 134 | H | -6.3500322 | -3.0056451 | 6.6462862 |

**Table S2.** Computed total energies of **1,7-i** and **1,6-i** at B3LYP/6-31G in Gas Phase.

| **1,7-i** | |
| --- | --- |
| Imaginary Freq | 84 |
| Temperature | 298.15 Kelvin |
| Pressure | 1 atm |
| Frequencies scaled by | 1 |
| Electronic Energy (EE) | -4109.8959 Hartree |
| Zero-point Energy Correction | 1.056996 Hartree |
| Thermal Correction to Energy | 1.104854 Hartree |
| Thermal Correction to Enthalpy | 1.105798 Hartree |
| Thermal Correction to Free Energy | 0.980166 Hartree |
| EE + Zero-point Energy | -4108.8389 Hartree |
| EE + Thermal Energy Correction | -4108.791 Hartree |
| EE + Thermal Enthalpy Correction | -4108.7901 Hartree |
| EE + Thermal Free Energy Correction | -4108.9157 Hartree |
| E (Thermal) | 693.306 kcal/mol |
| Heat Capacity (Cv) | 204.319 cal/mol-kelvin |
| Entropy (S) | 264.416 cal/mol-kelvin |

| **1,6-i** | |
| --- | --- |
| Imaginary Freq | 82 |
| Temperature | 298.15 Kelvin |
| Pressure | 1 atm |
| Frequencies scaled by | 1 |
| Electronic Energy (EE) | -4109.9354 Hartree |
| Zero-point Energy Correction | 1.056602 Hartree |
| Thermal Correction to Energy | 1.106077 Hartree |
| Thermal Correction to Enthalpy | 1.107021 Hartree |
| Thermal Correction to Free Energy | 0.976847 Hartree |
| EE + Zero-point Energy | -4108.8788 Hartree |
| EE + Thermal Energy Correction | -4108.8294 Hartree |
| EE + Thermal Enthalpy Correction | -4108.8284 Hartree |
| EE + Thermal Free Energy Correction | -4108.9586 Hartree |
| E (Thermal) | 694.074 kcal/mol |
| Heat Capacity (Cv) | 208.006 cal/mol-kelvin |
| Entropy (S) | 273.975 cal/mol-kelvin |

**
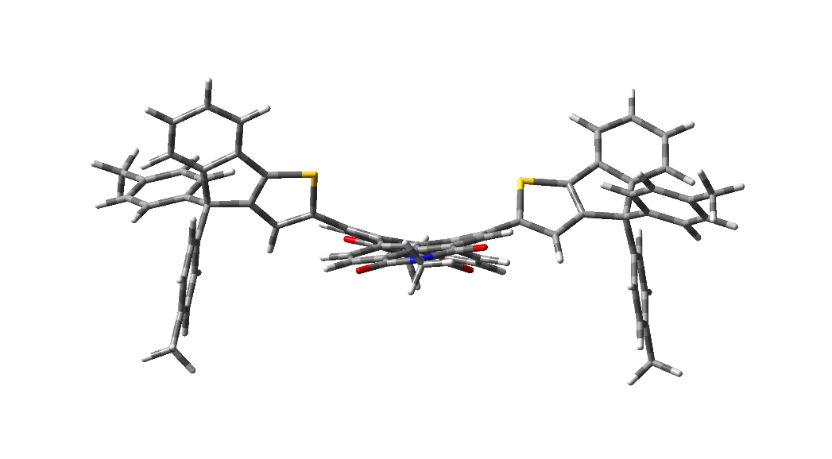

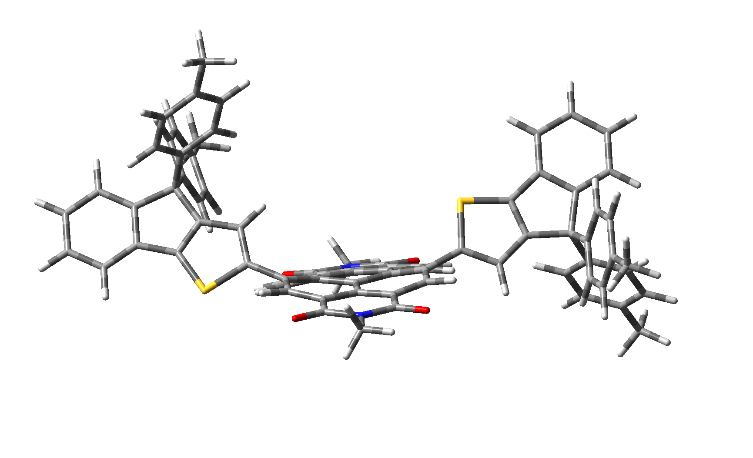
**

**1,7-i**

**1,6-i**

**Figure S17.** DFT optimized Geometry.

**Figure S18.** TD-DFT simulated spectra.

**Table S3.** Contribution to optical transitions obtained from TD_DFT calculations.

| Compound | λ_maxABS_ | Oscillator Strength | Major contribution | Minor contribution |
| --- | --- | --- | --- | --- |
| 1,6-i    1,7-i | @732  @522  @746  @513 | 0.18  0.37  0.30  0.43 | HOMO->LUMO  HOMO-2->LUMO  HOMO->LUMO  HOMO-2->LUMO | -  -  -  - |

**Photoelectron Spectroscopy in Air (PESA)**


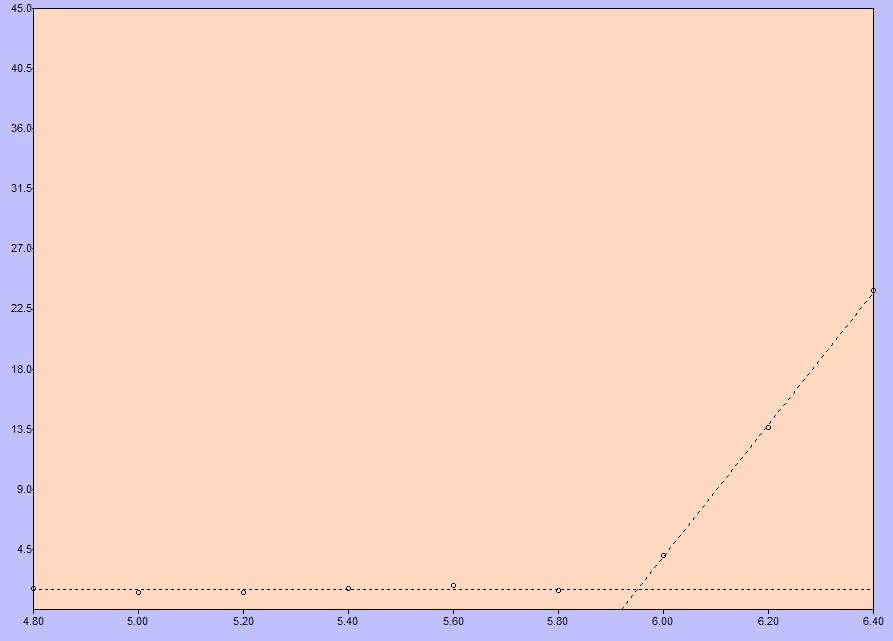


**1,6-i**

**-5.95 eV**


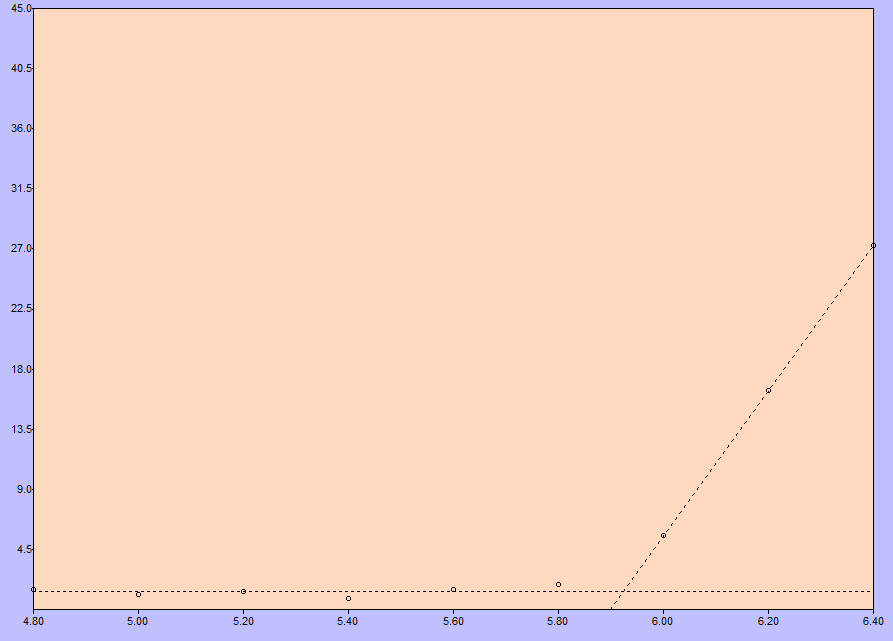


**1,7-i**

**-5.92 eV**

**Figure S19.** Photoelectron Spectroscopy in Air (PESA). Measured work function of **1,6-i** and **1,7-i** on spun-cast thin films, are in agreement with values obtained from CV or DFT calculation, showing a deeper HOMO for the *1,6* isomer.

**Crystal Structure Data**

**Table S4.** Crystal Data Collection and Refinement Parameters for **1,6-i** and **1,7-i.**

|  | **1,6-i** | **1,7-i** |
| --- | --- | --- |
| Empirical formula | C_84_H_66_N_2_O_4_S_2_, 2(C_7_H_8_) | C_84_H_66_N_2_O_4_S_2_, CH_2_Cl_2_ |
| Formula weight | 1415.77 | 1316.43 |
| Temperature (K) | 150.0(4) | 200.0(4) |
| Wavelength(Å) | 1.54184 | 1.54184 |
| Crystal system, Space group | Triclinic, P -1 | Monoclinic, I 2/a |
| a(Å) | 11.0190(7) | 31.0138(5) |
| b(Å) | 17.1710(9) | 18.5555(3) |
| c(Å) | 21.2015(15) | 50.4047(15) |
| α(deg) | 106.992(5) | 90 |
| β(deg) | 101.104(6) | 91.954(2) |
| γ(deg) | 91.781(5) | 90 |
| Volume (Å^3^) | 3748.2(4) | 28989.9(11) |
| Z | 2 | 16 |
| Calculated density (g cm^-3^) | 1.254 | 1.206 |
| Absorption coefficient(mm^-1^) | 1.087 | 1.748 |
| θ range (deg); completeness | 2.703-72.936; 98.1 % | 2.775-77.138; 96.0 % |
| Data collected / unique / R(int) | 30873 / 14316 / 0.071 | 60732 / 28211 / 0.037 |
| Data observed / parameters | 8942 / 953 | 22120 / 1699 |
| GOF | 1.03 | 1.096 |
| *R*_1_ / w*R_2_* [I > 2σ (I)] | 0.0791 / 0.1911 | 0.0719 / 0.2139 |
| *R*_1_ / *wR_2_* [all data] | 0.1216 / 0.2277 | 0.0857 / 0.2407 |
| Largest diff. peak and hole | 1.242 and -0.425 | 0.748 and -0.442 |
| CCDC number | 1955647 | 1955648 |


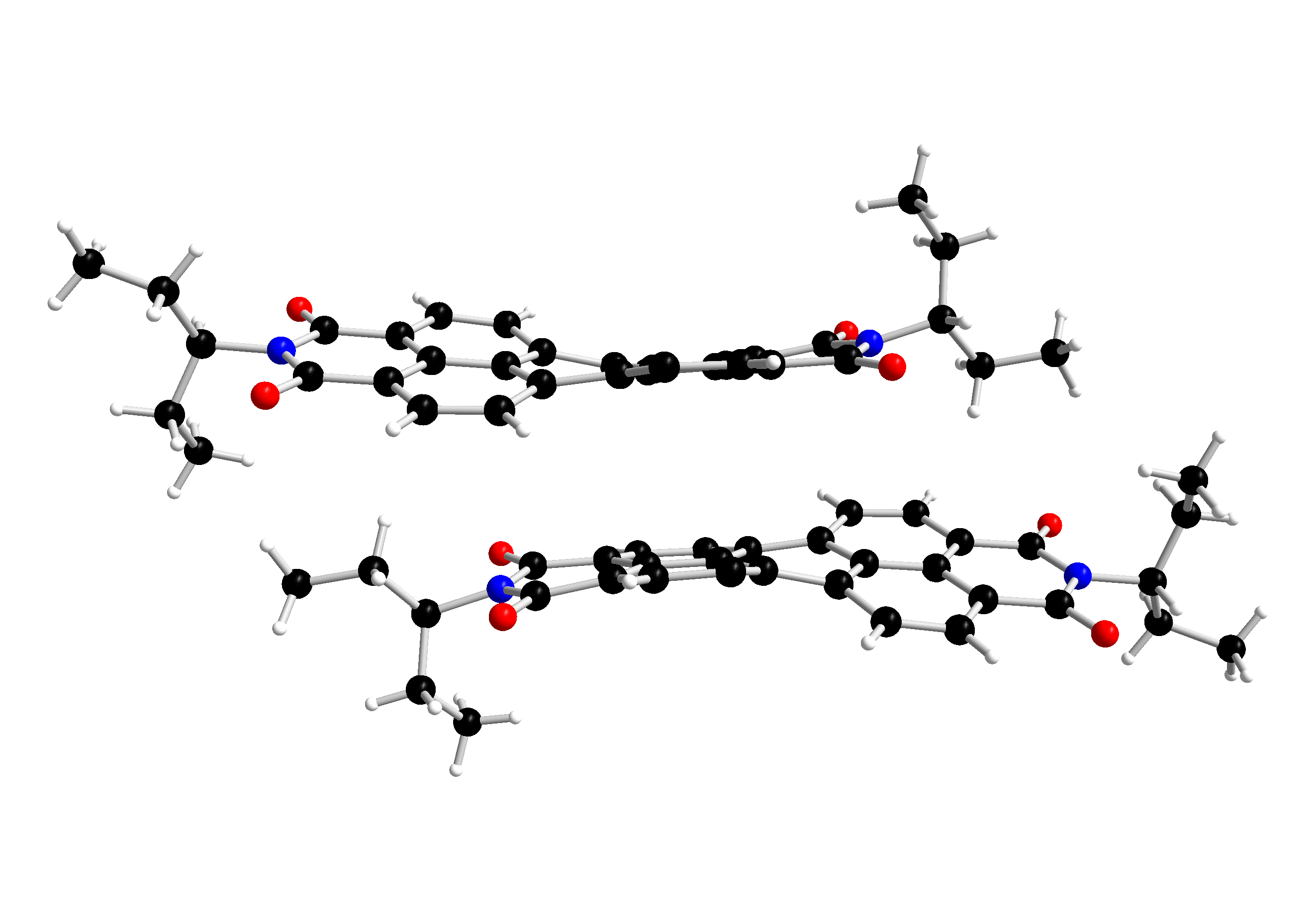


**Figure S20.** Perylene-perylene dimer stacking (**1,6-i**). IDT groups are omitted for clarity.


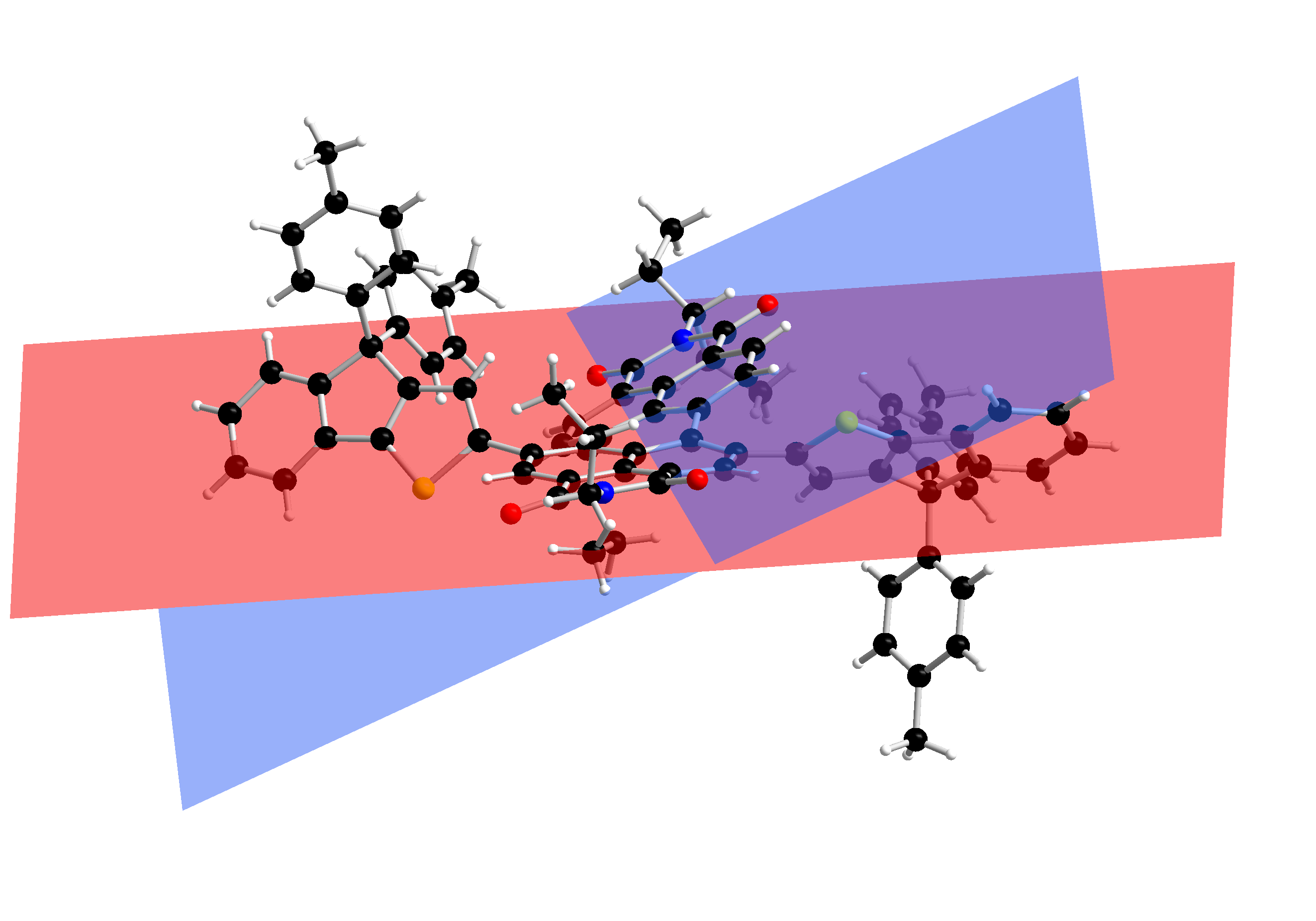


**Figure S21.** Subplanes of **1,6-i** single molecule.


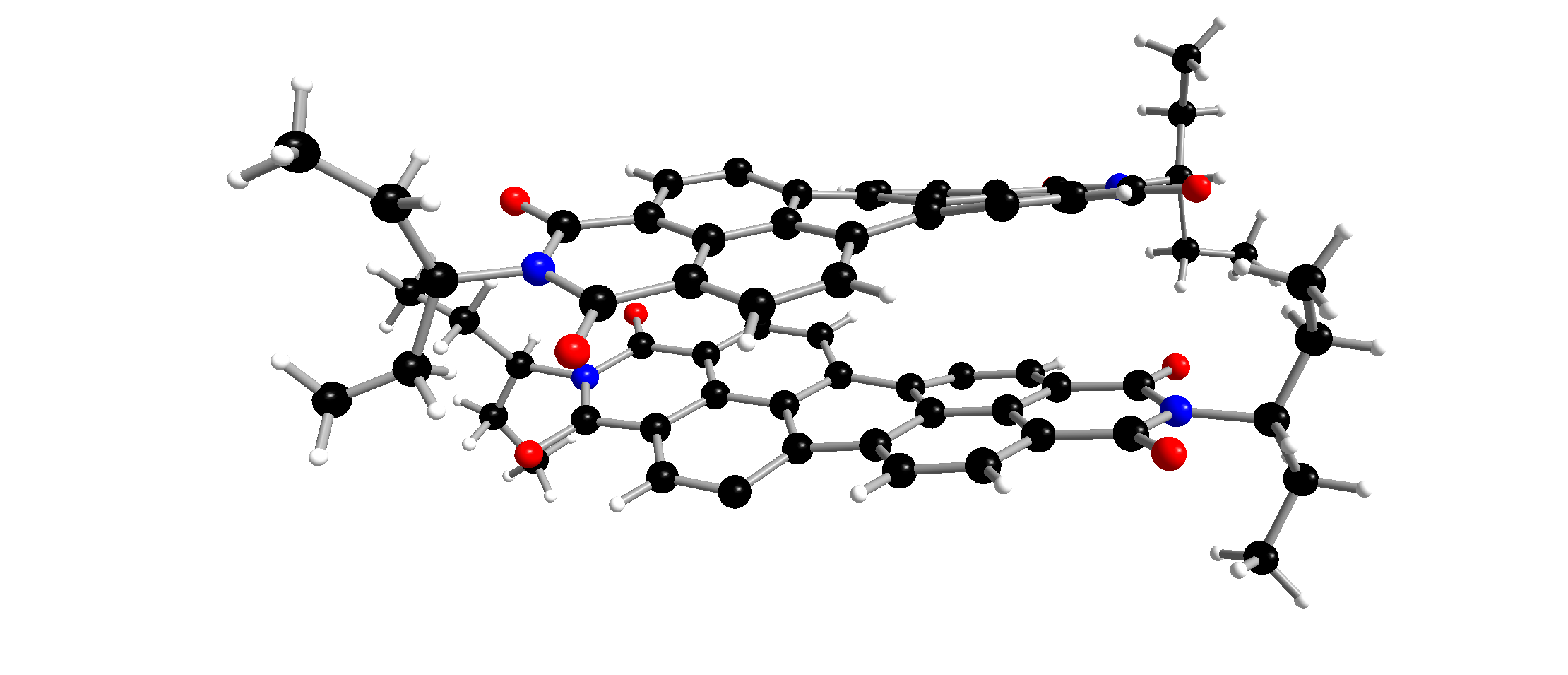


**Figure S22.** Perylene-perylene dimer stacking (**1,7-i**). IDT groups are omitted for clarity.


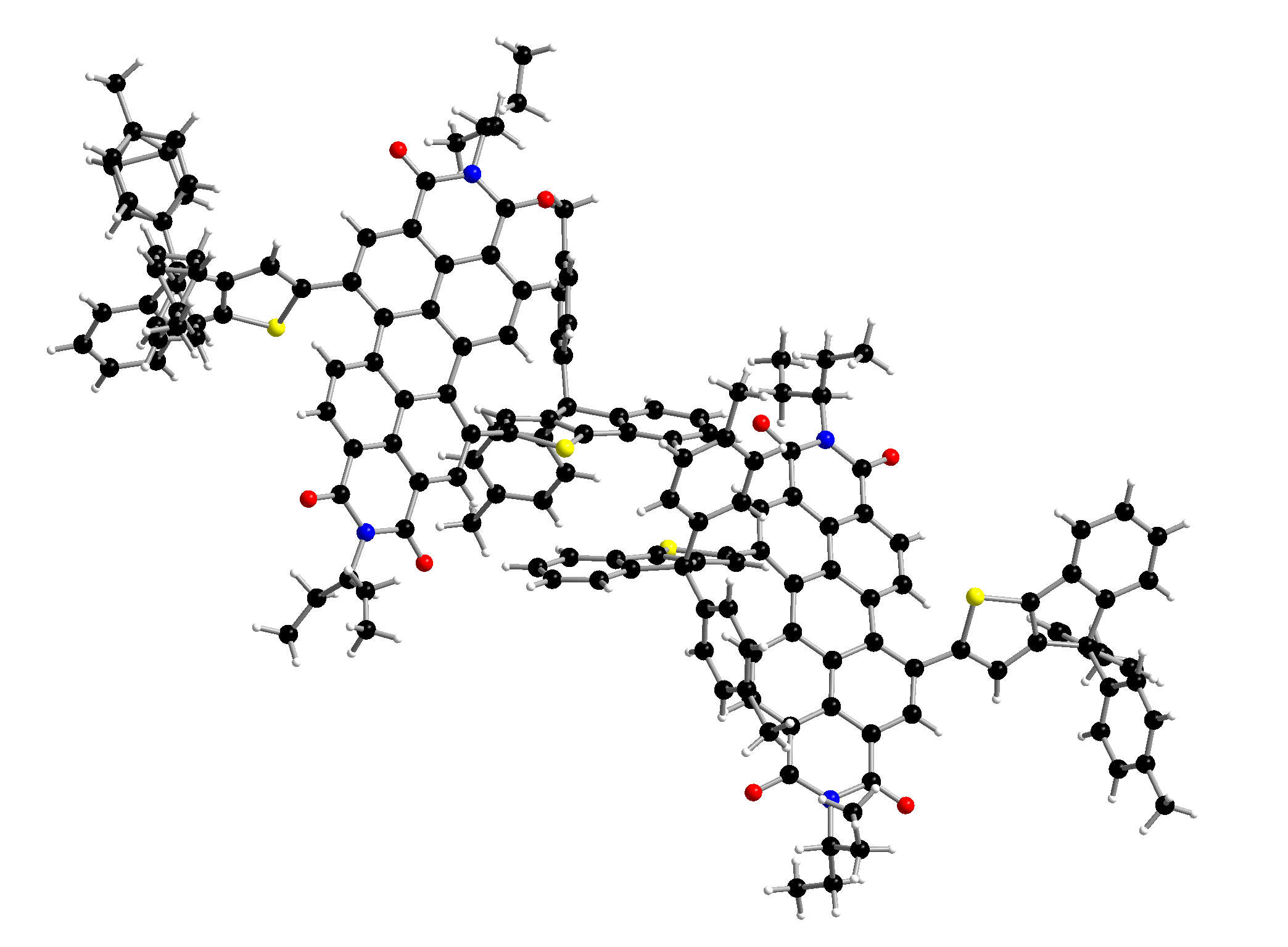
 **Figure S23.** IDT-IDT dimer stacking (**1,7-i**).
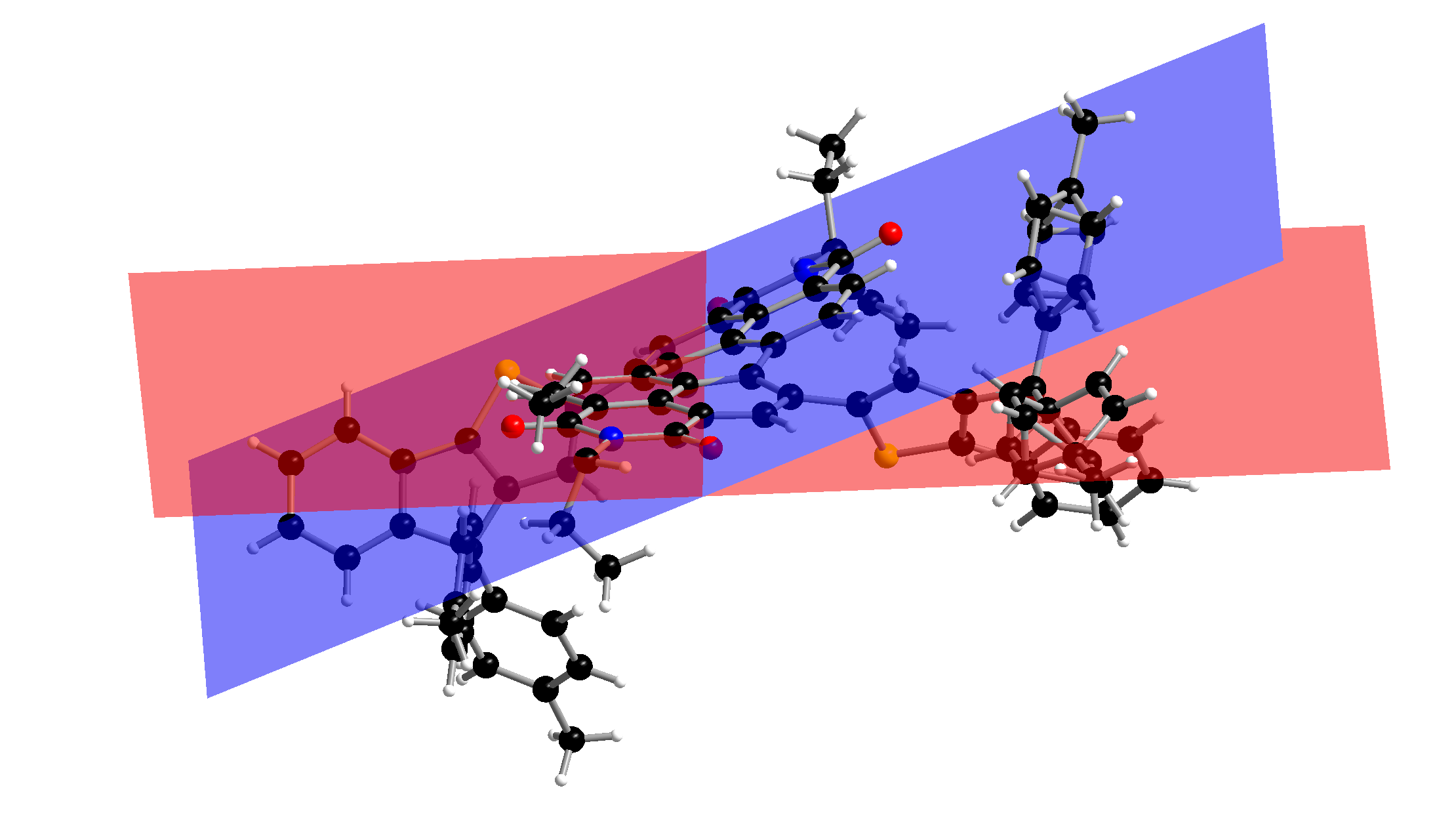


**Figure S24.** Subplanes of **1,7-i** single molecule.

**Photovoltaic data**

**Table S5:** photovoltaic parameters of devices made from pure to mixtures of isomers. Active layers were prepared from solution in Chlorobenzene at 10 mg/mL total concentration, with a polymer donor (PTB7-Th) : acceptor w/w ratio of 1:1. All values in the table are average values.

| **1,6-i** : **1,7-i** Ratio | V_oc_ (V) | J_sc_ (mA/cm^2^) | FF % | PCE % |
| --- | --- | --- | --- | --- |
| 10:0 | 0.86 | 4.7 | 41 | 1.6 |
| 9:1 | 0.89 | 4.1 | 39 | 1.4 |
| 7:3 | 0.89 | 3.2 | 37 | 1.0 |
| 5:5 | 0.89 | 2.7 | 35 | 0.8 |
| 3:7 | 0.90 | 2.4 | 33 | 0.7 |
| 1:9 | 0.89 | 2.3 | 33 | 0.7 |
| 0:10 | 0.89 | 2.4 | 33 | 0.7 |
